# Sub-Saharan Africa's Contribution to Clinical Trials in International Acute Coronary Syndromes and Heart Failure Guidelines

**DOI:** 10.1016/j.jacadv.2024.101383

**Published:** 2024-12-26

**Authors:** Jonathan A. Hudson, Leah Sanga, Modou Jobe, Anthony O. Etyang, David McAllister, Pablo Perel, Anoop SV. Shah, Elijah N. Ogola

**Affiliations:** a Kings College London BHF Centre, School of Cardiovascular and Metabolic Medicine & Sciences, London, United Kingdom; b Department of Non-communicable Disease Epidemiology, London School of Hygiene & Tropical Medicine, London, United Kingdom; c Medical Research Council Unit The Gambia at London School of Hygiene and Tropical Medicine, Fajara, The Gambia; d Department of Epidemiology and Demography, KEMRI-Wellcome Trust Research Programme, Kilifi, Kenya; e School of Health and Wellbeing, University of Glasgow, Glasgow, United Kingdom; f Department of Cardiology, Imperial College NHS Trust, London, United Kingdom; g Department of Clinical Medicine and Therapeutics, University of Nairobi, Nairobi, Kenya

**Keywords:** acute coronary syndrome, guidelines, heart failure, sub-Saharan Africa

## Abstract

**Background:**

There is a growing burden of acute coronary syndrome (ACS) and heart failure (HF) in sub-Saharan Africa (SSA), yet outcomes remain poor compared to high-income countries. The European Society of Cardiology (ESC) international guidelines are pivotal to the delivery of evidence-based care; however, their representation of populations from SSA remains unclear.

**Objectives:**

The purpose of the study was to evaluate the representation of populations from SSA in randomized controlled trials (RCTs) that inform ESC ACS and HF guidelines.

**Methods:**

We systematically analyzed pharmacotherapeutic RCTs contributing to the 2021 ESC HF and 2023 ACS guidelines, extracting data from ClinicalTrials.gov. We assessed the proportion of RCTs that included contributions from countries in each World Bank income group, focusing on the involvement of SSA countries and examining temporal trends.

**Results:**

Among the RCTs underpinning the ESC HF guidelines (n = 119) and ACS guidelines (n = 343), 75.9% were conducted exclusively in high-income countries. Middle-income countries were included in 22.2% of the trials, but none featured low-income countries. Within SSA, only South Africa was represented, contributing to 14.2% of HF and 8.2% of ACS RCTs. The number of HF RCTs involving populations from SSA has risen, from 2.6% in the 1990s to 50% post-2020 (*P* for trend< 0.05). For ACS RCTs, the proportion of trials involving populations in SSA increased from 1.8% pre-1990 to 23.4% during 2000 to 2009 (*P* for trend = 0.003), then declined to 11.3% in the following decade.

**Conclusions:**

There is a marked underrepresentation of SSA countries in ACS and HF pharmacotherapy RCTs. South Africa is the sole contributor from the region, which may affect applicability and generalizability of global guidelines to populations in SSA.

The escalating prevalence of cardiovascular diseases (CVDs) in sub-Saharan Africa (SSA) presents a significant public health challenge. Increasing life expectancy and changes in lifestyles have led to a growing prevalence of cardiovascular risk factors.^1^ Inadequate control of these via primary prevention leads to complications, particularly acute coronary syndrome (ACS) and heart failure (HF). This trend is reflected in regional SSA data, which indicates that CVD complications account for up to one-third of medical admissions.^2^

The prognosis from ACS and HF remains poor in the region, with SSA patients typically admitted when they are a decade younger yet experiencing case-fatality rates 2 to 3 fold higher compared to high-income countries (HICs).^3^^,^^4^ While HICs have made considerable progress in improving outcomes from acute cardiac disease in recent decades,^5^ a similar trajectory remains to be seen in SSA.

Evidence-based clinical guidelines have become a cornerstone of modern cardiovascular practice. By evaluating and summarizing available evidence, they assist health care professionals in proposing the best therapeutic approach for patients with a given condition. Globally accepted guidelines, predominantly developed by institutions in HICs, often set the standard for patient management. International guidelines to inform management of cardiac pathologies rely largely on clinical trial data generated in HICs. The generalizability of this data to SSA remains uncertain for several reasons. First, SSA populations may have distinct risk factor profiles from those in HICs, for example, high rates of associated infectious diseases such as HIV and tuberculosis.^6^ Second, the etiologies of diseases may differ, particularly with regard to HF. SSA has a higher prevalence of rheumatic heart disease and a much lower prevalence of ischemic cardiomyopathy.^7^ Third, the challenges faced by health care systems in SSA with regard to both the cost of cardiovascular interventions and access to care call into question the feasibility of delivering many clinical trial interventions in the region. Fourth, differential responses to pharmacotherapy may exist between different ethnicities.^8^^,^^9^

In view of these differences, we aimed to evaluate the extent to which populations from SSA contribute to pharmacotherapeutic randomized controlled trials (RCTs) informing international ACS and HF clinical guidelines.

## Methods

### Search strategy and eligibility criteria

We evaluated the 2023 European Society of Cardiology (ESC) guidelines for the management of ACS and the 2021 ESC guidelines for the diagnosis and treatment of acute and chronic HF.^10^^,^^11^ These were chosen as the 2 most widely used guidelines globally for the management of ACS and HF.^12^ All RCTs referenced in the guidelines were reviewed by 2 authors (J.H. and L.S.). We also reviewed all cited systematic reviews and meta-analyses for relevant RCTs. Studies were included if they were RCTs evaluating pharmacotherapeutic interventions contributing to Class I or II guideline recommendations.

### Data collection and analysis

The National Clinical Trial number was extracted from each RCT and used to extract trial data from the Aggregate Analysis of ClinicalTrials.gov (AACT) Database using R.^13^ Data extracted from the AACT database included intervention assessed, participating countries, the number of participating countries from SSA, and the total trial population enrolled. Lead author and year of publication were extracted from the citations. For trials that were not registered on ClinicalTrials.gov, data were extracted from the published article. If information on the participating country was not available from the AACT Database or the published article, other sources were interrogated including the International Standard Randomised Controlled Trial Number database, drug evaluations, and published secondary analyses.

The primary outcome was the proportion of studies that included a trial site in SSA. Further analyses were undertaken to summarize the number of trials by country-level gross domestic product per capita, country population, and World Data Bank derived country income status.^14^ Age-adjusted CVD mortality rates were extracted from the 2019 Global Burden of Disease.^15^ These were then plotted against the number of RCTs per country, which were extracted from the guidelines. This was stratified by World Data Bank-derived country income status. Countries were classified by region according to the Global Burden of Disease super region classification, and the World Bank definition of SSA was used.

A time trend analysis was performed by comparing the proportion of trials conducted in delineated time periods using a test for trend in proportions. Odds ratios (ORs) were derived by constructing 2 × 2 tables and using the number of clinical trials conducted pre-1990 as the reference for ACS and 1990 to 1999 for HF. All statistical analyses were conducted in R.

## Results

### Overview of clinical trials included in the review

A total of 657 studies were assessed for inclusion (Supplemental Figure 1). After screening, a total of 119 clinical trials from the ESC ACS guidelines and 343 RCTs from the ESC HF guidelines were included in the final analysis. These RCTs informed class 1 or 2 pharmacotherapeutic recommendations. Of the 462 RCTs, 351 (75.9%) were exclusively conducted in HICs. Of the RCTs, 103 (22.2%) had at least 1 upper middle-income country involved in the trials, while 51 trials (11%) had at least 1 lower middle-income country involved. There were no RCTs conducted in low-income countries. Supplemental Figure 2 shows the contribution of countries to the number of RCTs relative to their age-standardized CVD mortality and total population size.

#### Sub-Saharan African involvement

**Figure 1:**
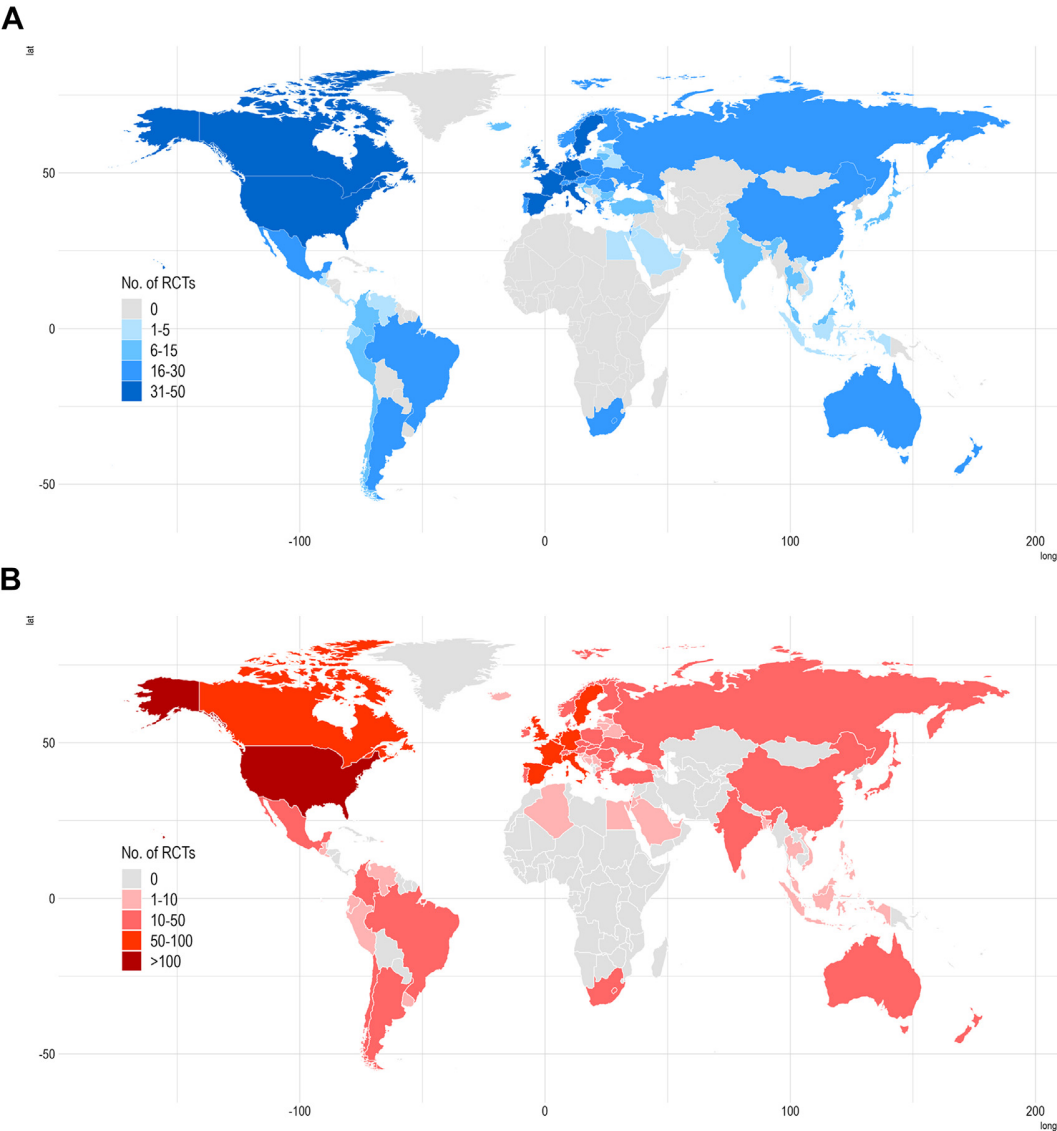
**Global Distribution of Pharmacotherapy Randomized Controlled Trials** The choropleth map demonstrates the distribution of pharmacotherapy randomized controlled trials that inform the (A) 2021 ESC guidelines for the diagnosis and treatment of acute and chronic heart failure and (B) 2023 ESC guidelines for the management of acute coronary syndromes. RCT = randomized controlled trial.

Of the 462 pharmacotherapy RCTs from both guidelines, 45 (9.7%) had a research site in an SSA country (Central Illustration). Of the 119 RCTs included from the ESC HF guidelines, 17 (14.2%) of these had at least 1 research site in a SSA country (Figure 1A). Of the 343 pharmacotherapy RCTs included from the ESC ACS guidelines, 28 (8.2%) of these had at least 1 research site in a SSA country (Figure 1B). The only SSA country represented in pharmacotherapy RCTs across both guidelines was South Africa.

**Figure 2:**
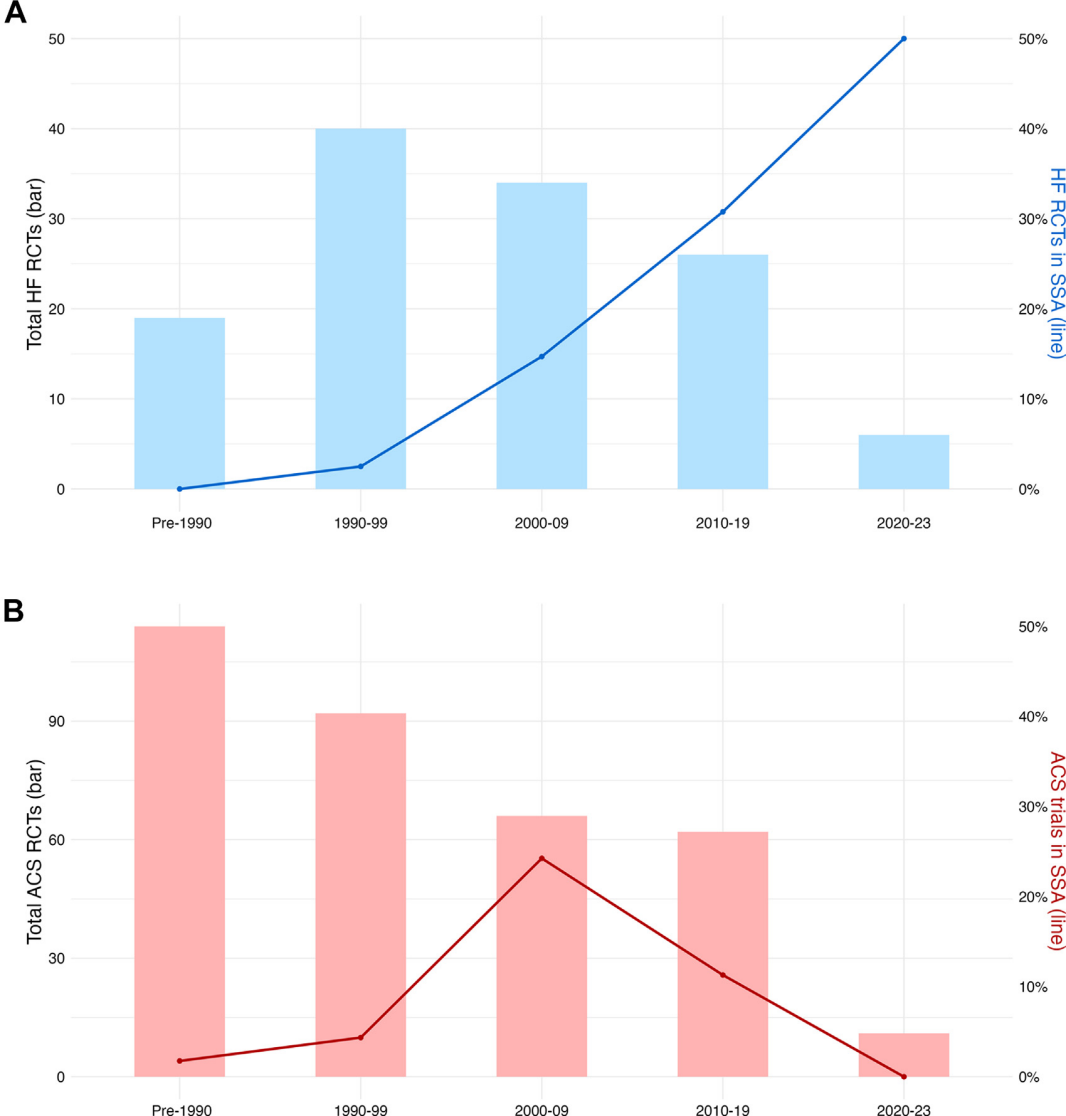
**Temporal Trends in Pharmacotherapy Randomized Controlled Trials Conducted in Sub-Saharan Africa** (A) Temporal trends in pharmacotherapy randomized controlled trials conducted in sub-Saharan Africa over time in the 2021 ESC guidelines for the diagnosis and treatment of acute and chronic heart failure and (B) the 2023 ESC guidelines for the management of acute coronary syndromes. The number of HF RCTs in SSA has risen, from 2.6% in the 1990s to 50% post-2020 (reference group: pre-1990, *P* for trend < 0.001). For ACS RCTs, the proportion of trials conducted in SSA increased from 1.8% pre-1990 to 23.4% during 2000-2009 (reference group: pre-1990, *P* for trend = 0.003) then declined to 11.3% the following decade. ACS = acute coronary syndrome; HF = heart failure; RCT = randomized controlled trial; SSA = sub-Saharan Africa.

The proportion of all HF and ACS RCTs conducted in SSA has risen over time (Figure 2). There were no HF trials conducted in SSA before 1990. Since then, the proportion has increased over time from 2.6% (n = 1/38 reference group) in 1990 to 1999, to 34.8% (n = 8/23) from 2010 to 2019 (OR: 16.8, 95% CI: 2.9-451.3), and 50% (n = 3/6) from 2020 to the present (OR 28.8, 95% CI: 2.6-983.4, *P* for trend < 0.001). For ACS, the proportion of trials that included populations from SSA pre-1990 were 1.8% (n = 2/114, reference group). This proportion peaked at 23.4% (n = 15/64, OR: 15.9, 95% CI: 4.2-112.9) in the period 2000 to 2009 and then dropped to 11.3% (n = 7/62, OR: 6.7, 95% CI: 1.5-50.9) from 2010 to 2019 (*P* for trend = 0.003).

**Central Illustration:**
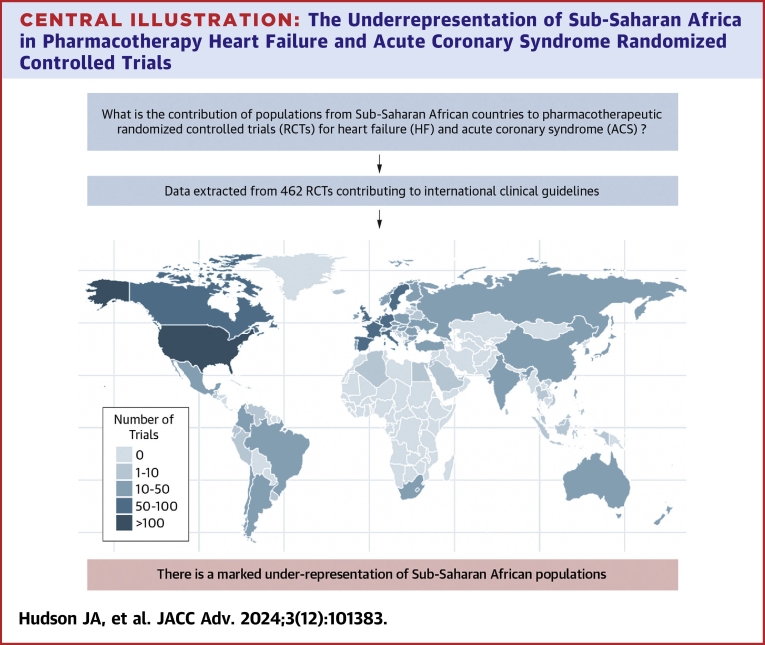
The Underrepresentation of Sub-Saharan Africa in Pharmacotherapy Heart Failure and Acute Coronary Syndrome Randomized Controlled Trials

When analyzed by the type of pharmacotherapy assessed in RCTs, for HF, 68% of mineralocorticoid receptor antagonist trials and 68% of sodium/glucose cotransporter 2 inhibitor trials included research sites in South Africa compared to 14.3% of renin angiotensin-aldosterone system inhibitors, 9.1% of beta blocker trials, and 0% of diuretic trials (Table 1). For ACS trials, 36.4% of renin angiotensin-aldosterone system inhibitor trials, 7.2% of the antithrombotic trials, 2% of beta blocker trials, and 15.4% of the lipid lower trials were conducted in SSA (Table 2).

**Table 1:** Heart Failure Randomized Controlled Trials Represented by Pharmacotherapy Type and Global Burden of Disease Super Region

	RAAS	Beta-Blockers	MRA	SGLT2i	Diuretics	Other
All RCTs	21	11	3	6	17	61
Sub-Saharan Africa	3 (14.3%)	1 (9.1%)	2 (66.7%)	4 (66.7%)	0 (0%)	7 (11.5%)
High income	21 (100%)	11 (100%)	3 (100%)	6 (100%)	17 (100%)	55 (90.2%)
Central Europe, Eastern Europe, and Central Asia	5 (23.8%)	6 (54.5%)	2 (66.7%)	6 (100%)	0 (0%)	17 (27.9%)
Southeast Asia, East Asia, and Oceania	3 (14.3%)	0 (0%)	0 (0%)	6 (100%)	0 (0%)	12 (19.7%)
South Asia	2 (9.5%)	0 (0%)	1 (33.3%)	5 (83.3%)	0 (0%)	5 (8.2%)
North Africa and Middle East	2 (9.5%)	3 (27.3%)	1 (33.3%)	4 (66.7%)	0 (0%)	14 (23%)
Latin America and the Caribbean	2 (9.5%)	1 (9.1%)	3 (100%)	6 (100%)	0 (0%)	14 (23%)

Values are n or n (%). Several trials were multicenter and therefore conducted in multiple regions.

MRA = mineralocorticoid receptor antagonist; RAAS = renin-angiotensin-aldosterone system; RCT = randomized controlled trial; SGLT2i = sodium/glucose cotransporter 2 inhibitor.

**Table 2:** Acute Coronary Syndrome Randomized Controlled Trials Represented by Pharmacotherapy Type and Global Burden of Disease Super Region

	Antithrombotic	RAAS	Beta Blockers	Lipid Lowering	Other
All RCTs	167	11	98	52	15
Sub-Saharan Africa	12 (7.2%)	4 (36.4%)	2 (2%)	8 (15.4%)	2 (13.3%)
High income	151 (90.4%)	10 (90.9%)	92 (93.9%)	43 (82.7%)	15 (100%)
Central Europe, Eastern Europe, and Central Asia	25 (15%)	4 (36.4%)	4 (4.1%)	11 (21.2%)	5 (33.3%)
Southeast Asia, East Asia, and Oceania	11 (6.6%)	1 (9.1%)	2 (2%)	6 (11.5%)	1 (6.7%)
South Asia	9 (5.4%)	0 (0%)	3 (3.1%)	2 (3.8%)	1 (6.7%)
North Africa and Middle East	13 (7.8%)	2 (18.2%)	1 (1%)	4 (7.7%)	3 (20%)
Latin America and the Caribbean	21 (12.6%)	4 (36.4%)	0 (0%)	7 (13.5%)	2 (13.3%)

Values are n or n (%).

Abbreviations as in Table 1.

## Discussion

This analysis of international HF and ACS guidelines has 3 key findings. First, SSA is the most underrepresented region globally in pharmacotherapy RCTs, which inform these guidelines. Second, although we show that there has been an increase in the proportion of RCTs being conducted in the region, the only country represented is South Africa. No trials included populations from Eastern, Western, or Central SSA. Third, HICs contribute the majority of the evidence, which informs guidelines with poor representation from lower middle-income countries and the complete absence of low-income countries.

There is an urgent need for the inclusion of SSA populations in CVD trials. Cardiovascular disease is the second leading cause of death in SSA and makes up 13.1% of all deaths.^16^ ACS and HF patients, in particular, suffer from high case fatality rates in the region.^4^^,^^17^ Despite being almost a decade younger, HF patients in Africa have mortality rates much higher than other global regions.^7^ For ACS, the data from SSA remains sparse.^18^ However, studies conducted in typical SSA settings have recorded 1 year mortality after acute myocardial infarction as high as 59.9%.^19^ Despite high disease burden and disproportionately high mortality rates, our analysis shows that most clinical trial data does not include these high-risk populations.

While analysis shows an encouraging increase in the representation of SSA populations in HF RCTs over the last 2 decades, this proportion is only based on a total of 6 trials post-2020. ACS trials also saw a more modest increase in SSA representation over the same period. These findings complement a recent bibliometric review by Noubiap et al,^20^ which demonstrated an increase in the annual volume of cardiovascular research from Africa. However, this increase represents only 3% of global cardiovascular research output.^20^ Reflecting our results, the authors also observed that South African research was the primary driver of this increase. This has not occurred accidentally. South Africa contributes the highest proportion of gross domestic product to support research in the region.^14^ Between 2001 and 2008, it more than doubled its gross expenditure on research and development, which led to a rapid rise in research output with a concurrent doubling in publications.^21^ Although South Africa’s highly developed private health care infrastructure may explain some of its success, it may also be considered as an example of how SSA countries can increase their research infrastructure and output.^22^

Poor research capacity and infrastructure have been cited historically as a reason for the lack of RCTs in SSA.^23^ This, however, ignores many widely cited and important RCTs that have shaped HIV and tuberculosis therapies that have been successfully conducted across several SSA countries.^24^, ^25^, ^26^ Furthermore, there are several recent, influential, and sophisticated CVD RCTs conducted in the region.^27^^,^^28^ Given that most pharmacotherapy trials in CVD are industry led by multinationals from the global north, the lack of financial incentive and reluctance to invest in the region may explain the low number of RCTs rather than a supposed lack of capacity.

The absence of almost all SSA countries from HF and ACS trials has significant implications for the treatment of these conditions in the region. Patients with HF from SSA are considerably younger and have a diverse range of etiologies that are context specific.^29^ The efficacy of guideline-directed medical therapy in SSA remains therefore largely unknown. In addition, SSA suffers from some of the most underinvested and weakest health care systems in the world.^22^ Secondary care infrastructure for the delivery of HF and ACS treatment is very poor.^30^ How best to implement evidence-based recommendations such as supporting the titration of HF pharmacotherapy remains unknown. Future research will require a contextualized system-based approach considering the wider context in which health care is delivered, including social, cultural, and health system factors.

Several interventions should be considered to address the under representation of SSA populations in CVD RCTs. First, increased national and charitable investment in noncommunicable disease research is essential for the region.^31^ Second, it is necessary to strengthen research capacity and infrastructure, including the expansion of clinical trial units and the establishment of robust training programs.^32^ Third, developing collaborative networks and partnerships is crucial; these should include both intraregional collaborations among SSA countries and partnerships with global academic centers. Fourth, pharmaceutical companies should be encouraged to conduct clinical trials within SSA. Finally, robust regulatory frameworks are needed in the region. It is important that researchers and organizations ensure that all research conducted is contextually relevant and culturally appropriate.

### Study limitations

While our analysis brings insights into the representation of SSA populations in HF and ACS pharmacotherapy trials, we recognize certain limitations. First, many studies did not provide the participant number by country, preventing us from providing an accurate reflection of the population proportion by region in the trial. Global trials must be encouraged to report participant numbers by country so that differences between global regions can be scrutinized.^33^ Second, there may be RCTs that do include populations from SSA or low-income countries but do not contribute to clinical guidelines. Third, our focus was on the ESC guidelines and did not include other major guidelines. Fourth, restricting the analysis to RCTs may neglect other study designs that could provide meaningful insights into the treatment of CVDs in SSA.

## Conclusions

This analysis demonstrates a marked underrepresentation of SSA countries in ACS and HF pharmacotherapy RCTs, despite the region's escalating CVD burden. South Africa is the sole contributor from the region, with all other SSA countries absent. This finding could potentially affect the applicability and generalizability of guidelines to a population with distinct clinical presentations and health care challenges. For meaningful advancements in cardiovascular care in SSA, there is an imperative to invest in regionally representative research.

Perspectives **COMPETENCY IN MEDICAL KNOWLEDGE:** The underrepresentation of SSA populations in clinical trials for ACS and HF limits the relevance of current guidelines to SSA patients. Region-specific research is critical to ensure that guidelines reflect the unique clinical challenges and health care realities in SSA.

**TRANSLATIONAL OUTLOOK:** Greater investment in SSA research infrastructure and capacity is crucial to include SSA populations in global CVD trials and for conduct of specific trials contextualized to the region. Addressing barriers such as limited financial incentives and perceived research capacity will require fostering international collaborations and conducting region-specific RCTs to adapt guidelines to SSA contexts.

## Funding support and author disclosures

Dr Hudson is funded by the NIHR (NIHR Academic Clinical Fellowship [ACF-2021-17-001]). The project is funded by 10.13039/100006662NIHR Award Number 134544. The authors have reported that they have no relationships relevant to the contents of this paper to disclose.
