# Supplementary material for: Sub-Saharan Africa's Contribution to Clinical Trials in International Acute Coronary Syndromes and Heart Failure Guidelines
Source: JACC Adv. 2024 Dec 26;3(12):101383. doi: 10.1016/j.jacadv.2024.101383 (PMC11734027; doi:10.1016/j.jacadv.2024.101383)

## Supplemental Figure 1: PRISMA flow chart of trials identification, screening, and inclusion


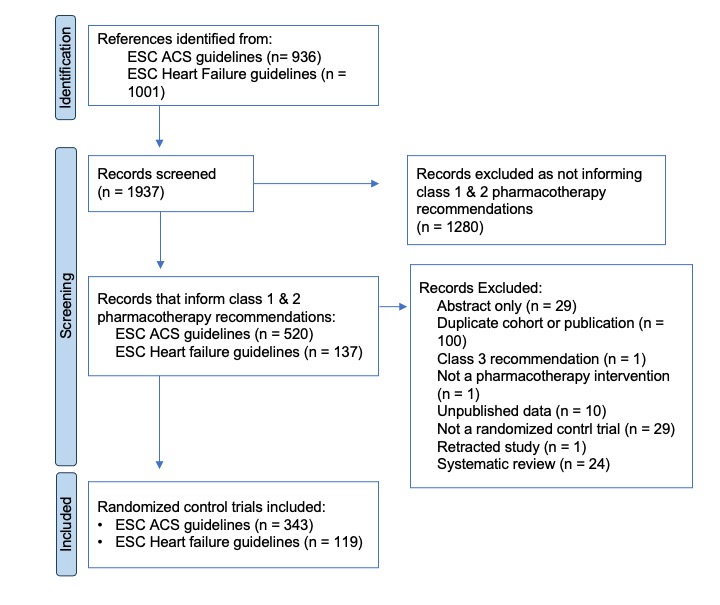


## Supplemental Figure 2: Scatter Plot of the Number of Clinical Trials Versus Age-Standardized Cardiovascular Disease Mortality Rates by Country, Differentiated by Income Level, with Circle Size Proportional to Population Size


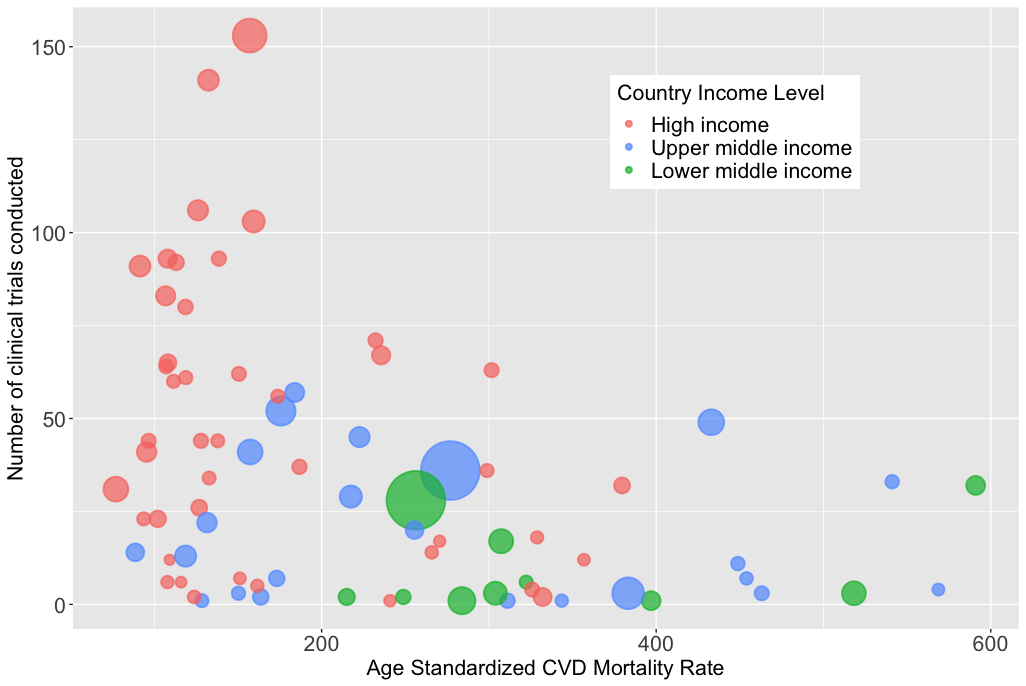

Supplement: Supplemental material [file mmc1.docx]
